# Dynamic analysis of towed cable with variable length during turning maneuvers

**DOI:** 10.1038/s41598-023-30731-8

**Published:** 2023-03-02

**Authors:** Dapeng Zhang, Bowen Zhao, Keqiang Zhu, Haoyu Jiang

**Affiliations:** 1 Ship and Maritime College, Guangdong Ocean University, Zhanjiang, 524088 Guangdong China; 2 Ocean College, Zhejiang University, Zhoushan, 316000 Zhejiang China; 3 Faculty of Maritime and Transportation, Ningbo University, Ningbo, 315211 Zhejiang China; 4 School of Electronics and Information Engineering, Guangdong Ocean University, Zhanjiang, 524088 Guangdong China

**Keywords:** Environmental sciences, Ocean sciences

## Abstract

The configuration of marine towing cable changes significantly during the turning process, with the rotating procedure with fixed cable length being the most frequent. To overcome these challenges, the configuration and dynamic properties of the marine towing cable must be addressed. However, under some particular operating situations, the tugboat must release the marine towed cable during rotation, resulting in a constant change in the length of the marine cable. In view of this, the towed cable is discretized into a lumped mass model based on the lumped mass method, and the dynamic analysis model of the rotation process of towed cable with variable length under different release speeds and different depths is established. This is done with reference to the specific parameters of a towed system, combined with the specific sea conditions of a particular sea area. Time-domain coupling analysis is used to determine the dynamic changes in configuration and stress of marine towing cable at various release speeds and depths. The results of the calculations have some guiding relevance for a certain engineering practice.

## Introduction

Ocean towing systems are becoming more and more crucial in a variety of disciplines, including ocean monitoring, military detection, seabed mapping, and naval defense, as ocean development progresses^1–3^. The systems in many different sorts of applications include the towing ship, towed cable, and towed body. The towed system often performs a variety of motions while operating, including linear acceleration, turning, and zig-zagging^4–7^. The steady-state studies of Choo and Casarella^8^ are primarily responsible for our current understanding of how an underwater tow line is configured during ship maneuvering. Chapman^9^ identified three types of dynamic cable behavior: a gradual turn with a low towed speed and a large turning radius to cable length ratio, a sharp turn with a high towed speed and a small turning radius R to the cable length L ratio R/L, and the transient state between two turns. For a given towing speed, he determined a critical ship-turning radius above which the cable/vehicle system maintains an equilibrium shape that is almost equivalent to the planar configuration associated with a straight-ahead-towing ship trajectory. The towing mechanism effectively fails below the required turning radius. A full turning maneuver instance was utilized as an illustration and validation of the practicality of their nonlinear dynamic modals by Ablow and Schechter^10^ Milinazzo et al.^11^, Gobat and Grosenbaugh^12^, and many others.

Many researchers are now investigating the dynamic behavior of undersea cables during ship maneuvers. For instance, Kishore and Ganapathy^13^ simulated the behavior of towed array during a full circle turning course. The case studies were carried out for different loop radii, towed lengths, towing speeds and truncated loops. It was found that reducing the towing speed during a loop from 9.85 m/s to about 3.5 m/s cause the tow point tension to drop down sharply. The fast relaxation in tow-point tension might lead to severe problems in the onboard end of the cable. Grosenbaugh^14^ examined the dynamic behavior of a towed cable system that results from the towing ship changing course from a straight-tow trajectory to one involving steady circular turning at a constant radius. Buckham et al.^15^ and Lambert et al.^16^ developed a mathematical/computational model of a towed underwater vehicle system and discussed an application of the model to improve the performance of the system during a turn maneuver. The mathematical model was connected with non-linear numerical simulations of an autonomous surface vehicle and an actively managed towed fish. The lumped mass approximation was used to simulate the towed cable. The results showed that the towed underwater vehicle simulation could be used in an optimization algorithm to find the optimum balloon turn geometry for a U-turn maneuver. Wang and Sun^17^ parametrically simulated the dynamic response of a towed cable system to ship maneuver. The influence of three dimensionless parameters on towed cable system maneuverability was investigated, ratio of total length to turning radius, ratio of cable mass to vehicle mass, and ratio of mass unit length to hydrodynamic force. The results showed that the transient behavior between two small equal radii circle turnings revealed a growing turning effect on the gradual turn which dominates horizontal trajectory. Zhang et al.^18^ studied the dynamic characteristics of fixed length cables under the turning state of the towing ship. The bending changes of the cables were found to be more frequent and severe in the vicinity of 15 m towards the head and tail ends. Zhao et al.^19^ developed a full-coupled three-dimensional dynamic model of a towed cable body system. Numerical simulations were conducted for different maneuvers, including the straight and U-turn tows. The numerical simulation and the sea trial data were compared, and the findings revealed that the numerical simulation and the sea trial data were in good agreement. Using a numerical technique, Yuan et al.^20^ estimated how an underwater towed system would affect the maneuverability of the towing ship. It was discovered that the towing ship's velocity, turning radius, and roll angle were lowered throughout the turning maneuver. Zhang et al.^21^ studied the dynamic response of a cable-towed system during ship in 180 degrees U-turn maneuver. A numerical model of marine cables with bending stiffness was presented based on three-dimensional lump-parameter approach and validated by OrcaFlex.

The dynamic behavior of a towing cable in a turn, according to the literature, might be seen as a trade-off between the period of the turn and the decay duration of the transient. A lengthy time period determines a steady cable system profile in a turn with a big radius^22,23^. Instead, a slow decay period results in a sharp decline in depth. However, the knowledge that can be acquired from earlier research concerning the cable transient behavior is not sufficiently thorough. The transient behavior of the towing cable system during various ship maneuvers is poorly understood. For example, when the tugboat is laying the submarine pipeline, the tugboat will release the cable while turning^24^. During ship turning maneuvers, when the cable length fluctuates, it is uncertain that how towing cable systems would behave^25,26^. In this paper, the towed cable is discretized into a lumped mass model based on the lumped mass method with reference to the specific parameters of a towed system in conjunction with the specific sea conditions of a particular sea area. A dynamic analysis model of the rotation process of a marine cable with variable length under different release speeds and different depths is then established. The dynamic changes of configuration and tension of marine cable under different release speeds and different depths are obtained by time-domain coupling analysis.

## The theoretical model

### Static balance calculation method

**Figure 1:**
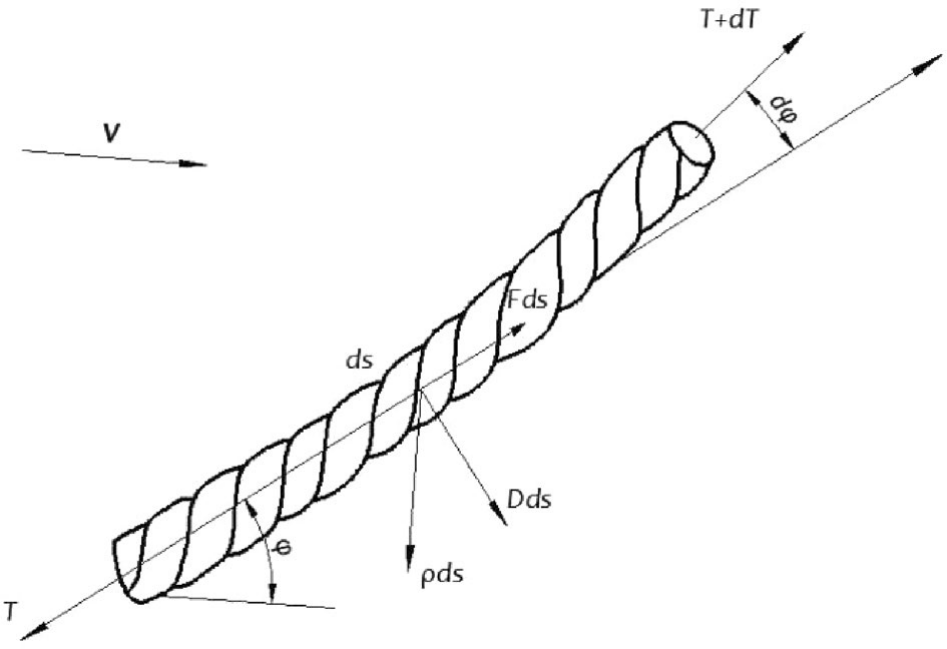
Diagram of cable micro-element force.

In the ocean, the cable is a typical thin, flexible component. The thin flexible components in the ocean must first achieve static balance and take on their static balance form before dynamic simulation could commence. As a result, the static balance step must come first. The catenary method, which is briefly described here, is used to calculate the static balance of offshore thin flexible components. As shown in Fig. 1, *ds* is a certain micro-element on the cable, *D* and *F* are the fluid forces per unit length along the vertical and tangential directions of the cable element, respectively. *T* is the tension of the cable. *φ* is the angle between the cable element and the water flow direction, which is called the cable angle. *dT* and *dφ* are the small increments of the tension on the cable element *ds* and the cable angle *φ*, respectively. *w* is the underwater weight of the cable per unit length.

In the normal direction of the cable element:

1 $$ Td\varphi - \rho gA(h - z)d\varphi = (w\cos \varphi + D)ds $$

where, *ρ* is the fluid density, *g* is the gravity of cable micro-element, *A* is the sectional area of cable micro-element, *h* is water depth, *z* is seabed depth.

In the tangent direction of the cable element:

2 $$ dT - \rho gAdz = (w\sin \varphi - F)ds $$

The apparent tension T* = *T* − *ρgA*(*h *− *z*) is introduced, the above equations can be written as following:

3 $$ T^{*} d\varphi = (w\cos \varphi + D)ds $$

4 $$ dT^{*} = (w\sin \varphi - F)ds $$

For the sake of brevity, the apostrophe * on the *T* is omitted.

The above two cable balancing equations are nonlinear, and analytical solutions are difficult to discover. Under certain conditions, it is possible to seek a simpler analytical solution. If the cable is heavy or the current velocity is small, the force acting on it is mostly gravity, and the fluid force may be neglected. Equations (3) and (4) can be simplified as follows:

5 $$ Td\varphi = w\cos \varphi ds $$

6 $$ dT = w\sin \varphi ds $$

Divide (5) and (6):

7 $$ \ln \frac{T}{{T_{0} }} = \mathop \smallint \limits_{{\varphi_{0} }}^{\varphi } \tan \varphi d\varphi = \ln \sec \varphi = \ln \frac{{\cos \varphi_{0} }}{\cos \varphi } $$

Hence:

8 $$ T = T_{0} \frac{{\cos \varphi_{0} }}{\cos \varphi } $$

where, *T* is the tension of the cable when the cable angle $\varphi_{0}$ is 0.

Substitute Eq. (8) into Eq. (5), and measure from the origin point, integrate from the cable length *s*_0_ to the cable length *s* (set the cable angles at the two places to be *φ*_0_ and *φ* respectively), the following Eq. (9) can be gotten:

9 $$ s - s_{0} = \mathop \smallint \limits_{{\varphi_{0} }}^{\varphi } \frac{{T_{0} \cos \varphi_{0} }}{{w\cos^{2} \varphi }}d\varphi = \frac{{T_{0} \cos \varphi_{0} }}{w}(\tan \varphi - \tan \varphi_{0} ) $$

Let *T*_*h*_ = *T*_0_cos*φ*_0_, hence:

10 $$ s - s_{0} = \mathop \smallint \limits_{{\varphi_{0} }}^{\varphi } \frac{{T_{0} \cos \varphi_{0} }}{{w\cos^{2} \varphi }}d\varphi = \frac{{T_{h} }}{w}(\tan \varphi - \tan \varphi_{0} ) $$

The following equation can be gotten by substituting *dx* = *dscosφ* into Eq. (5):

11 $$ s - s_{0} = \mathop \smallint \limits_{{\varphi_{0} }}^{\varphi } \frac{{T_{0} \cos \varphi_{0} }}{{w\cos^{2} \varphi }}d\varphi = \frac{{T_{h} }}{w}(\tan \varphi - \tan \varphi_{0} ) $$

Similarly, the Eq. (12) can be obtained by substituting *dz* = *dssinφ* into Eq. (5):

12 $$ s - s_{0} = \mathop \smallint \limits_{{\varphi_{0} }}^{\varphi } \frac{{T_{0} \cos \varphi_{0} }}{{w\cos^{2} \varphi }}d\varphi = \frac{{T_{h} }}{w}(\tan \varphi - \tan \varphi_{0} ) $$

Equations (11) and (12) are expressions between the cable length, the horizontal distance and the vertical distance at any two points in the static equilibrium stage of the cable. If the lower limit of the integral is taken at the origin point, then:

13 $$ \frac{w}{{T_{h} }}x = \ln \left( {\frac{1 + \sin \varphi }{{\cos \varphi }}} \right) $$

If *a* = *T*_*h*_/*w*, there are:

14 $$ \frac{x}{a} = \ln \left( {\frac{1 + \sin \phi }{{\cos \phi }}} \right) $$

It can be concluded that:

15 $$ \sinh \frac{x}{a} = \tan \phi $$

16 $$ \cosh \frac{x}{a} = \frac{1}{\cos \phi } $$

Thus, Eqs. (10) and (12) can be written as follows:

17 $$ s = a\sinh \frac{x}{a} $$

18 $$ z = a\left( {\cosh \frac{x}{a} - 1} \right) $$

Equations (17) and (18) are catenary equations.

From Eqs. (17) and (18), we can get:

19 $$ (z + a)^{2} = s^{2} + a^{2} $$

20 $$ s = \sqrt {z(z + 2a)} $$

21 $$ x = a\cosh^{ - 1} \left( {\frac{z}{a} + 1} \right) $$

This is how the classic catenary approach, which relies on the idea that fluid forces outweigh gravity, was developed. The geometry of the cable in the static equilibrium stage in this work is based on the aforementioned theory since this technique may yield a clear analytical answer, allowing it to be referred to as an analytical catenary method.

### Numerical method and validation

The numerical methods for cable analysis can be divided into three categories: finite element method, finite difference method and lumped mass method. The finite element method is frequently utilized in engineering calculations. When applied to the study of flexible risers, however, there will be more components and nodes as the length of the flexible riser rises. The order of stiffness of the matrix will rise as the number of elements and nodes increases, and the matrix will become extremely sparse. As a result, achieving the convergence of the computing process is challenging and necessitates a significant amount of computation time. A numerical approach for solving differential equations of subsea pipelines is the finite difference method with differential equations, including both ordinary and partial differential equations, are covered. The discrete approximation of the differential, which is necessary for the finite difference technique, employs the function values of the discrete points to estimate the differential of the point. The finite difference method's main advantage is that it is easy to understand and requires no creation of form functions. But it is not appropriate for engineering issues with intricate boundary conditions. The lumped mass method directly calculates from Newton's second law, approximating the cable as a series of nodes connected by massless linear elastic elements and applying distributed forces like gravity and fluid dynamics on the distributed nodes of the cable. This is in contrast to the finite difference method, which solves the governing equation from the point of view of micro-elements.

In this paper, the lumped mass method is used as a calculation method of umbilical cable. The idea of this method is to divide the cable into N segments, and the mass of each element is concentrated on one node. There are N + 1 nodes, whereas tension *T* and shear *V* acting on the ends of each segment can be concentrated on a node, and any external hydrodynamic load is concentrated on the node. The equation of motion of *i*-th node (*i* = 0, 1…N) is:

22 $$ M_{{A_{i} }} \ddot{R}_{i} = T_{{e_{i} }} - T_{{e_{i - 1} }} + F_{{dI_{i} }} + V_{i} - V_{i - 1} + w_{i} \Delta \overline{s}_{i} $$

where, *R* represents the node position of the cable.

$M_{Ai} = \Delta \overline{s}_{i} \left( {m_{i} + \frac{\pi }{4}D_{i}^{2} (C_{an} - 1)} \right)I - \Delta \overline{s}_{i} \frac{\pi }{4}D_{i}^{2} (C_{an} - 1)(\tau_{i} \otimes \tau_{i - 1} )$ is the mass matrix of a node, *I* is a 3 × 3 identity matrix; $T_{ei} = EA\varepsilon_{i} = EA\frac{{\Delta s_{0i} }}{{\Delta s_{\varepsilon i} }}$, which stands for effective tension at a certain node; $\Delta s_{0i} = \frac{{L_{0} }}{{\left( {N - 1} \right)}}$, which represents the original length of each segment; $\Delta s_{\varepsilon i} = \left| {R_{i + 1} - R_{i} } \right|$, the stretched length of each segment; *EA*, axial stiffness of the cable. $F_{{dI_{i} }}$ represents the external hydrodynamics of each node, which is calculated according to the Morison equation.

In order to verify the correctness of the lumped mass method, a towed cable is taken and moved under the specified boundary condition^27^. The calculation results are compared with the previous results. The towed cable includes cable, array and drogue. The properties of the towed cable are as shown in Table 1.

**Table 1:** Validation parameters.

Parameter	Cable	Array	Drogue
Length (m)	723	273.9	30.5
Mass per length (kg/m)	1.5895	5.07	0.58
Wet weight per length (N/m)	2.33	0	0.57
Diameter (m)	0.041	0.079	0.025
Axial stiffness EA (N)	1 × 10^8^	1 × 10^8^	5 × 10^6^
Bending stiffness EI (N m)	1000	1000	0.01
*ρ*_*w*_	2	1.8	1.8

**Figure 2:**
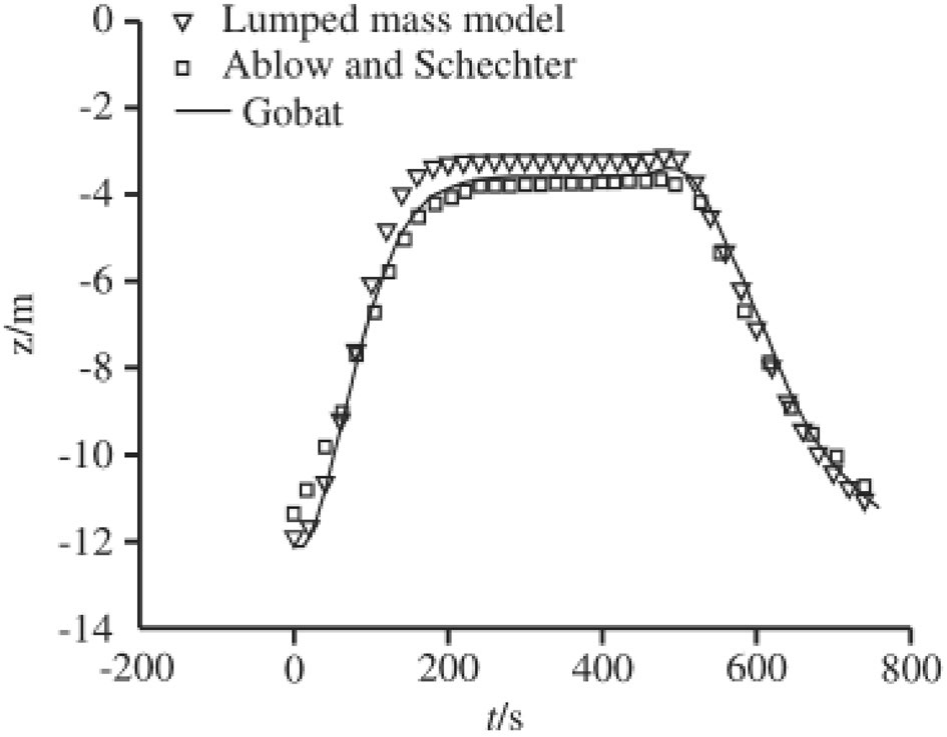
Validation for the numerical method.

A point located at 8.2 m of the array profile is selected and compared with the previous studies. The variations of depth are compared to the results of Gobat and Grosenbaugh^28^ and Ablow^10^. The findings of the lumped mass model are shown in Fig. 2 to be consistent with earlier research and to show that the minimum depth is more similar to the measured depth. All of the comparisons demonstrate that the lumped mass approach is quite precise.

## Numerical model set-up

The top end of the marine cable is connected to the tail of the tugboat in OrcaFlex, and the lower end is anchored on the seabed. The initial shape of the marine cable is catenary. The marine environment parameters are as follows: the sea water density is 1025 kg/m^3^, no wind or waves, and the current speed of 0 m/s. The marine cable parameters are: the outer diameter of the marine cable of 0.9652 m, the inner diameter of 0.9015 m, the Young's modulus is 22,000 Mpa, the Poisson's ratio is 0, the density is 7.85 t/m^3^, the normal added mass coefficient is 1, and the normal towed force coefficient is 0.8. Since the surface coating of the marine cable is smooth (the surface coating of the marine cable is 0.05715 m, and its density is 2.4 t/m^3^), the axial added mass coefficient and axial drag force coefficient are both 0. The normal friction coefficient between the marine cable and the seabed is 1, and the axial friction coefficient between the marine cable and the seabed is 0 because the marine cable does not run through the seabed.

The tugboat makes a direct motion with a speed of 1 m/s for the initial 20 s, and then the tugboat begins to make a turn motion with a duration of 900 s. The gyration angular velocity of the gyration tugboat is 0.2°/s, and the linear velocity of gyration tugboat is 1 m/s. After completing the 900 s gyration, the tugboat exited the gyration mode and performed a direct course for 500 s, still at 1 m/s in the direct course. The tip of the marine cable is connected to the tail of the tugboat and the initial length of the marine cable is 1950 m during release.

**Figure 3:**
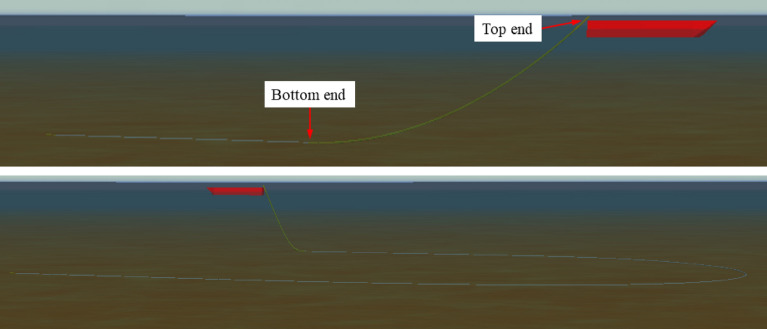
Model of marine cable with variable length during turning process.

When analyzing the influence of the release speed on the dynamic characteristics of the release process of the marine cable, the water depth is kept at 100 m, and the marine cable is released at the speed of 0.8, 0.9, 1.0, 1.1 and 1.2 m/s, respectively during the tugboat's rotation. The smoothing growth control factor was utilized to manage the smoothness of the towing cable's growth throughout the release process, and the lowest variable segment's growth factor was set to 0.001. When analyzing the influence of water depth on the dynamic characteristics of the segmentation of the marine cable, keep the release speed of the marine cable at 1 m/s, and set the water depth at 50, 75, 100, 125 and 150 m, respectively. The marine cable release process in the turning process of tugboat is shown in Fig. 3.

## Results and discussion

### Dynamic characteristics at different release speeds

**Figure 4:**
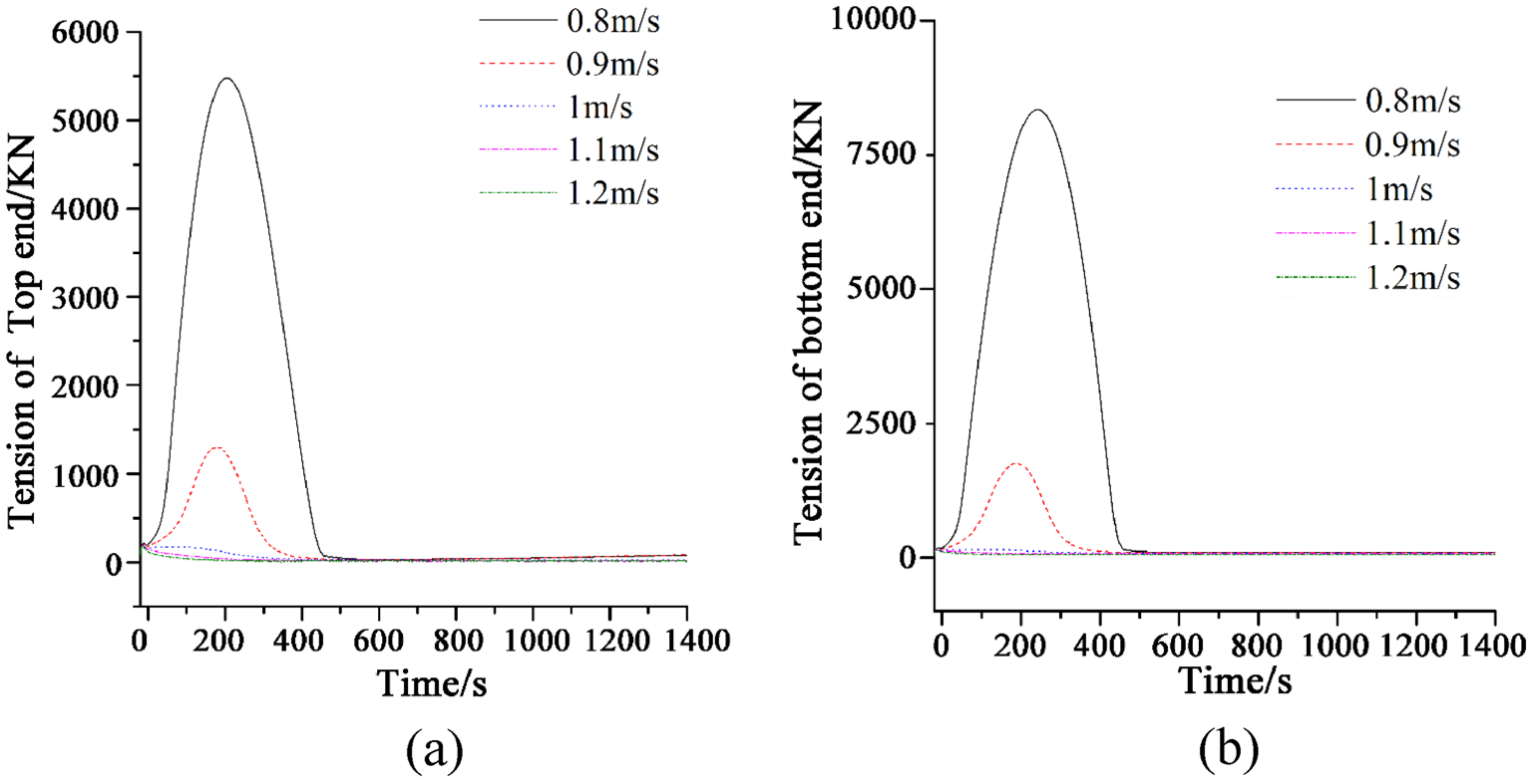
(**a**) Tension of the marine cable at top end at different release rates; (**b**) Tension of the marine cable at bottom end at different release rates.

Figure 4 shows the tension of marine cable at two ends with different release rates during turning process. The top end of the marine cable is connected to the tail of the tugboat, and the bottom end is in contact with the seabed. The maximum tension of the bottom end is greater than that of the top end. The reason for this phenomenon is that during the release process, the cable and the seabed are constantly rubbing. The rotation of the tugboat will also produce a drag force at the bottom of the cable. Therefore, the forces at the bottom end include its own gravity, the friction force on the seabed and the drag force.

It can be seen from Fig. 4 that, when the release speed of the cable is low (0.8–0.9 m/s), with the increase of the release speed, the maximum tension at the top and bottom of the cable will decrease. In the whole-time domain, the tension at both ends of the cable will increase first and then decrease in the range of 0–500 s, while the tension at both ends of the cable will decrease and stabilize at a lower value after 500 s. With the increase of the release speed (1–1.2 m/s), the fluctuation of the tension at the top and bottom of the cable in the time domain is significantly reduced, and the maximum tension at both ends of the cable is significantly reduced compared with that at low-speed release, and the time when the tension is stable is greatly advanced. The reason for this phenomenon is that when the release speed is low, the pulling effect caused by the turning of the tugboat and the self-weight of the cable is concentrated at both ends of the cable. However, with the acceleration of the release speed, the drag effect caused by the turning of the tugboat and the self-weight of the cable is released to some extent. Therefore, when the release speed is high, with the increase of the release speed, the maximum tension at both ends of the cable shows a significant downward trend. The reason is that, when the cable is released at a very fast speed, the axial tension of the cable caused by the tugboat's turning pulling and the cable's self-weight will be equal to (or even less than) the increased release of the cable at this time. Therefore, it can be understood that the cable at this time is basically not subject to tension, so the tension at both ends is extremely low. Further observation shows that the tension at both ends of the cable decreases significantly when the turning speed of the tugboat is equal to the release speed of the cable. In this case, the pulling elongation of the cable caused by the turning of the tugboat at each time step is approximately equal to the length increment of the cable. Therefore, when the release speed of the cable is the same as the linear speed of the tugboat, the cable will not stretch significantly during the whole turning process. When the release speed of the cable is lower than the turning speed of the tugboat, the maximum tension on the bottom of the cable is greater than the maximum tension on the top of the cable, which means that the tension on the bottom of the cable is greater than the top of the cable, when the release speed of the cable is lower than the turning speed of the tugboat.

**Figure 5:**
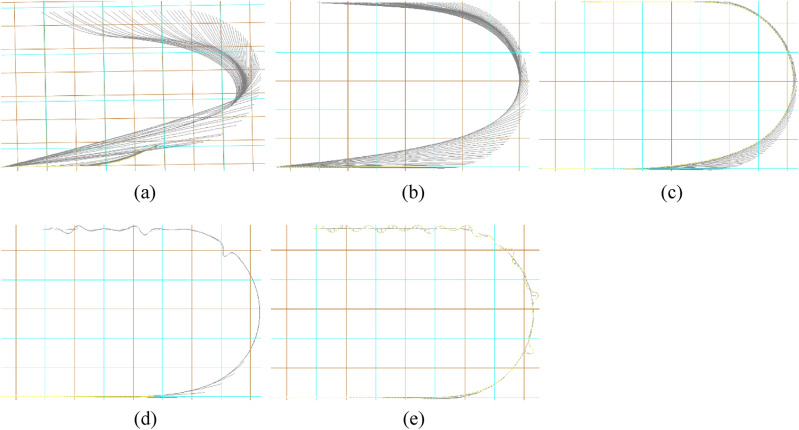
Configurations of marine cable at top view at different release rates during turning process: (**a**) 0.8 m/s; (**b**) 0.9 m/s; (**c**) 1.0 m/s; (**d**) 1.1 m/s; (**e**) 1.2 m/s.

Figure 5 depicts the configurations of marine cable at top view with different release speeds during turning process. In Fig. 5, the length of a small gridline is 50 m. The white line is the overhead projection of the cable at each time step during the turning process. Under the condition of constant water depth, if the release speed of the cable is too small, the cable is very easy to overstretch during the straight sailing and small angle turning phase of the tugboat. As the release speed approaches the tugboat speed, the trend of this overstretching of the cable will gradually weaken. When the released speed is the same as the turning speed, because the elongation of the cable at each time step is roughly the same as the total advance of the tugboat at each time step, the cable will not be stretched too much at this stage. As the release speed continues to increase and the turning process continues, the underwater suspension part of the cable will not be severely stretched at each time step. Most of the time, the cable is in a loose catenary shape and placed on the seabed. When the release speed exceeds the turning speed, the released length of the cable at each time step is greater than the forward displacement of the tugboat. With the increase of time step, the cable may have local bending, twisting and tangling. For simulation modeling, this will reduce the convergence of the whole system. In practical projects, local entanglement of the cable will lead to local wear and excessive bending stress of the cable. To sum up, the release speed of the marine cable should not be too small or too large, and not exceed the turning speed of the tugboat. Ignoring other factors, the release speed of the marine cable which is the same as the turning speed of the tugboat is the appropriate release speed of the marine cable.

### Dynamic characteristics at different water depths

**Figure 6:**
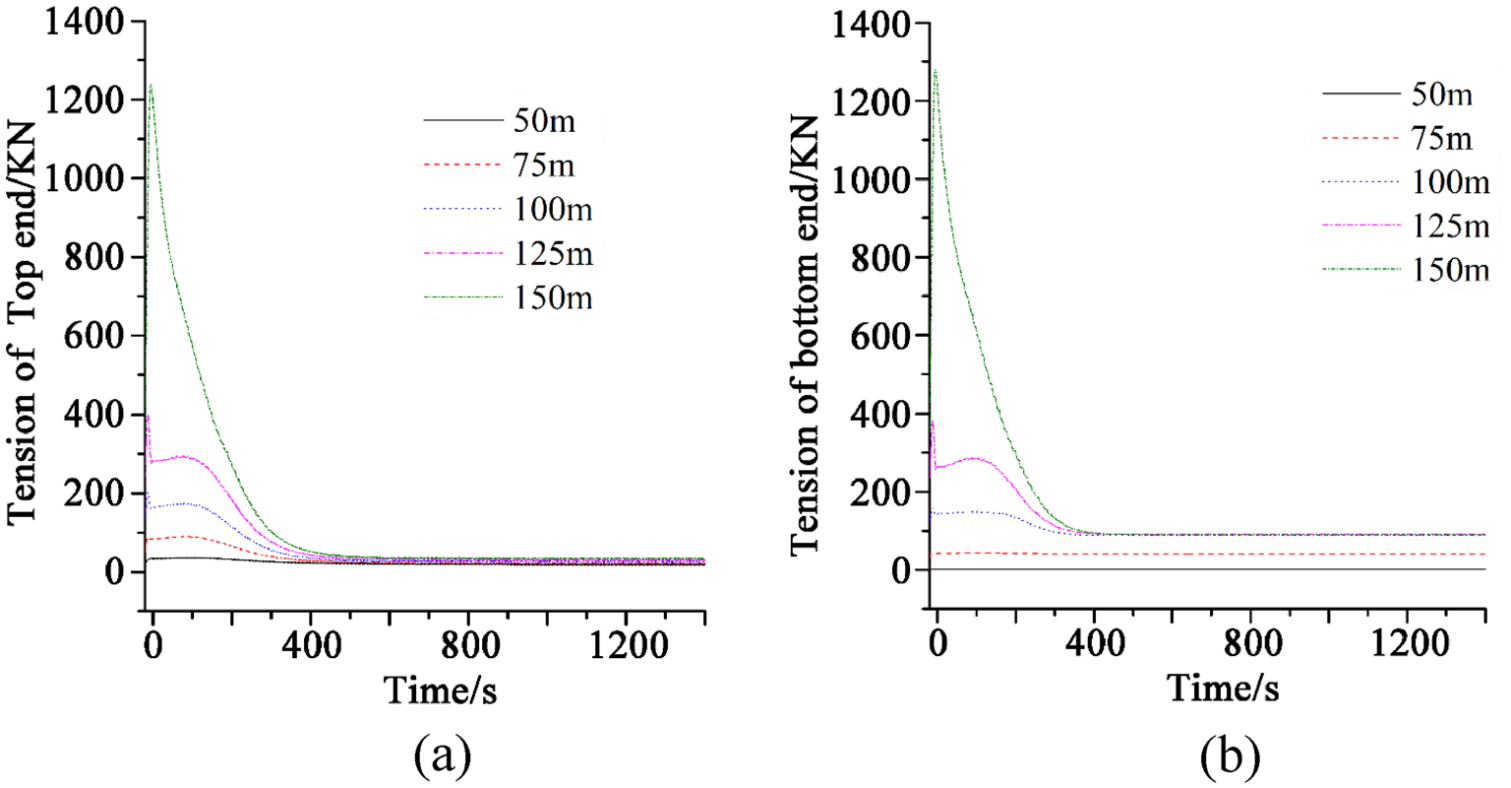
(**a**) Tension of the marine cable at top end at different water depths; (**b**) Tension of the marine cable at bottom end at different water depths.

The cable will not noticeably extend during the whole turning operation, as shown in "Dynamic characteristics at different release speeds", if the release speed of the cable is the same as the tugboat's turning speed. Therefore, the release speed of the cable in this part is equal to the turning speed of the tugboat, both of which are 1 m/s, in order to examine the impact of the water depth on the dynamic characteristics of the cable. Figure 6 depicts the tension of the marine cable at two ends throughout the turning operation at various water depths. The time domain pictures of stress at both ends of the cable at the same water depth exhibit a significant degree of resemblance when the tugboat's turning speed and the cable's release speed are both constant. The maximum tension on the cable at both ends will rise as the depth of the water does as well. The maximum tension of the cable is slightly higher at its top than it is at its lowest, though, when the sea is shallow (50–125 m).

Furthermore, when the water depth is between 0 and 50 m, the stress at the cable's bottom is practically constant throughout the whole-time domain. In the water depth range of 0–50 m, the released length of the cable at each time step can preserve the form of the bottom of the cable, resulting in a weak traction effect. At this point, the stress at the cable's bottom is almost constant. When the water depth is between 100 to 125 m, the tension at both ends of the cable first increases in the time domain and then decreases abruptly. The tension is basically unchanged within 0–200 s, decreases again in 200–500 s and remains unchanged after 500 s. When the water depth is 75 m below the intermediate water depth, the time domain variation of tension at both ends of the cable is entirely different. The tension of the tip cable remains constant in the time domain at first, then gradually lowers to a stable value. At this depth, the bottom tension does not fluctuate over time. This indicates that the turning process has a modest depth of water, which re-duces the tension variation at both ends of the cable. This is relevant to cable deployment and has a guiding meaning for actual engineering practice. Further investigation reveals that as water depth increases, the period for tension at both ends of the line to stay steady before dropping diminishes. When the water depth exceeds 150 m, the tension at both ends of the line quickly decreases after reaching its maximum. Further investigation indicated that the time for tension at both ends of the cable to stay steady before dropping reduced consecutively as water depth increased. When the water depth reaches 150 m, the tension at both ends of the line quickly drops after reaching its maximum.

**Figure 7:**
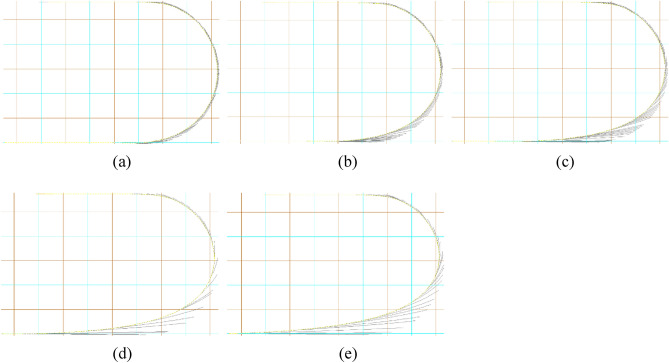
Configurations of marine cable at top view at different water depths during turning process: (**a**) 50 m; (**b**) 75 m; (**c**) 100 m; (**d**) 125 m; (**e**) 150 m.

Figure 7 depicts the configurations of marine cable at top view at different water depths during turning process. The white line is the overhead projection of the cable at each time step during the turning process. In Fig. 7, the length of a small gridline is 50 m. When the water depth is shallow, the cable will not be subjected to acute stress due to the tugboat's turning and continual re-lease of the cable. At this time, the cable's suspension section can maintain a certain catenary shape with the tugboat's advance, and the tugboat's advance will bring a limited increase in tension to the cable, which is conducive to reducing the bending deformation of the cable due to the self-weight of the suspension. When the water depth exceeds 100 m and the tug-boat turns forward, the suspension span section of the cable is greatly stretched at a given point of turning, and the draft suspension span section can no longer retain the catenary form all the time. When the water depth is shallow, the bending deformation of the suspension section of the cable at the initial moment decreases, the catenary becomes steeper and steeper, and the tensile stress in the process of rotation progressively increases. When the water depth rises to a particular level, the cable will be stretched to a great amount during the turning operation. This indicates that there is a threshold water depth for a certain cable release speed while maintaining the tugboat's linear velocity and radius of gyration constant. The cable will be heavily tugged at some point throughout the turning release procedure after the water depth is attained.

## Conclusion

The dynamic properties of a marine cable with changing length during turning maneuvers are explored in this work. The marine cable is discretized into a lumped mass model using the lumped mass method, and a dynamic analysis model of the turning process of the marine cable is established for different release speeds and water depths. This study demonstrated the following:

1. In the turning process, when the release speed of the cable is low, the maximum tension at the top and bottom of the cable will decrease. The pulling effect caused by the turning of the tugboat and the self-weight of the cable is concentrated at both ends of the cable. The tugging effect is lessened as the cable's release speed is increased. When the re-lease speed is high, there is a noticeable decreasing trend in the maximum tension at both ends of the cable as the release speed increases.
2. The tension at both ends of the cable decreases significantly when the turning speed of the tugboat is equal to the release-speed of the cable. The pulling elongation of the cable caused by the turning of the tugboat at each time step is approximately equal to the length increment of the cable. When the release speed of the cable is the same as the linear speed of the tugboat, the cable will not stretch significantly during the whole turning process.
3. The turning process has a reasonable water depth, which reduces tension fluctuation at both ends of the cable. The time it takes for the tension at both ends of the line to stabilize before descending reduces as the depth of the water increases.

## Data Availability

All data generated or analysed during this study are included in this published article. The datasets used and/or analysed during the current study available from the corresponding author on reasonable request.
